# The importance of the comparative benchmark for measuring composite financial literacy with survey data

**DOI:** 10.3389/fpsyg.2022.1025555

**Published:** 2022-12-07

**Authors:** Fang Liu, Hanyun Lei, Wenyi Lu, Qiang Yin

**Affiliations:** ^1^School of Finance, Xinjiang University of Finance and Economics, Ürümqi, China; ^2^School of Management, Jiangsu University of Technology, Changzhou, China; ^3^School of Finance, Zhejiang Gongshang University, Hangzhou, China; ^4^Research Division, Asian Growth Research Institute, Kitakyushu, Japan; ^5^Business School, Jishou University, Jishou, China

**Keywords:** comparative benchmark, composite financial literacy, objectively-measured financial literacy, subjectively-perceived financial literacy, overconfidence

## Abstract

**Introduction:**

Using survey data to calculate composite financial literacy (CFL), existed studies do not consider the geographical difference of the means of objectively-measured financial literacy and subjectively-perceived financial literacy, i.e., comparative benchmark.

**Methods:**

Taking the survey data of National Financial Capability Study (NFCS) for example, we explain why it is more reasonable to use the within-state average rather than the national average of financial literacy as the comparative benchmark to measure CFL. Then we use NFCS 2009, 2012, 2015 and 2018 dataset to comparatively analyze the difference between CFL measured with the two benchmarks.

**Results:**

The results of statistical analysis show that there is a great difference among the four groups of CFL measured with the two benchmarks, and 10.7% of respondents are categorized as a particular group of CFL incorrectly for all datasets. Additionally, the findings of spatial distribution analysis unveils that 36, 19, 15, and 6 states have respondents miscategorized in the four groups of CFL for 2009, 2012, 2015, and 2018 respectively, in which the highest proportion of the population miscategorized in a state is up to 49.91%. Finally, we find that several groups of CFL measured with the two benchmarks have significantly different effects on stock market participation behavior.

**Discussion:**

Using the national average as a benchmark to determine all the respondents’ relative financial literacy levels for different states is not meaningful, and will lose the practical appeal to tackle the regional inequalities of financial literacy among the households. Therefore, we suggest that the within-state average of financial literacy, not the national average, should be taken as the comparative benchmark for identifying the more precise groups of CFL in survey.

## Introduction

The 2008–2009 global financial crises attach more importance to individual’s financial literacy (FL) and its impact on household wealth. However, the lack of FL may lead to poor financial decisions and then decrease household financial welfare. Many studies have proved that this is evident for many countries (Bucher-Koenen and Lusardi, 2011; Agnew et al., 2012; Arrondel et al., 2013; Agarwal et al., 2015; Boisclair et al., 2017; Grohmann et al., 2018; Peng et al., 2020). While composite FL (CFL) combined objectively-measured FL (OFL) with subjectively-perceived FL (SFL) ^1^ also has a vital impact on financial behaviors (Allgood and Walstad, 2013).

To study the OFL’s interaction with SFL could be beneficial because it can further help us to understand how FL comprehensively affects individuals’ financial behaviors. As an original research, Allgood and Walstad (2013) develop a method, which can combine an individual’s actual financial knowledge with self-perceived financial knowledge. They prove that this combination could provide ‘more robust and nuanced insights’ about how FL affects credit card behaviors. Since the study of Allgood and Walstad (2013), many studies use the CFL measured with the their method as a significant independent variable to explain individuals’ or households’ stock market participation (Xia et al., 2014; Yeh and Ling, 2022), risky financial behaviors (Asaad, 2015), financial advice usage (Porto and Xiao, 2016), various financial behaviors (Allgood and Walstad, 2016), investment fraud victimization (Xiao et al., 2022), and stock investment return (Liao et al., 2022). They all discover that the CFL can provide more insights and show a greater effect on financial behaviors or outcomes than OFL alone. However, none of these studies concern about the rationality of the method developed by Allgood and Walstad (2013).

The method provided by Allgood and Walstad (2013) categorizes respondents as having high or low FL if their FL scores are above the overall, i.e., national average, and then obtains four CFL combinations: OFL-high and SFL-high, OFL-low and SFL-low, OFL-high and SFL-low, and OFL-low and SFL-high. A critical setting of this method is that the national average is used as the comparative benchmark to determine which respondent has high or low OFL or SFL. However, Peng et al. (2018) unveil that FL of American varies substantially across states and tends to cluster in space. We thus raise the concern that using the national average as the cutoff point to determine the high and low OFL or SFL of respondents in different states could neglect the potential geographical impact. For example, the highest within-state average of OFL is Montana 3.45, while the lowest within-state average of OFL is Florida 2.71 for 2015 dataset of National Financial Capability Study (NFCS). The gap between the two averages is 0.74, which is substantial, considering the overall scale of OFL is only 5.^2^ A respondent with OFL score 3.1 should be considered as relatively financial literate if he lives in Florida, while relatively financial illiterate if he lives in Montana. However, using the national average in 2015 (2.98) instead of the within-state average as a cut-off point, could categorize the respondent with OFL score 3.1 as the group with high FL whether he lives in Florida or Montana, or any other state. Therefore, using the average score at nation level could not exclude potential geographic impact. Furthermore, the essence of CFL is the relative confidence of respondents on FL, which is determined by the benchmarks of FL compared with. Obviously, the respondents only could compare their FL with the people nearby, not the people far away. In addition, respondents living in the same state have similar cultural environment, which is an important impact factor of confidence and the basis for the comparison of FL.

Therefore, it is more reasonable to replace national average with within-state average as comparative benchmark to measure CFL. Using the survey data of NFCS 2009, 2012, 2015 and 2018 to comparatively analyze the difference between CFL measured with SA method and NA method,^3^ we discover a considerable difference between the results of NA and SA method, not documented in previous research. Specifically, 10.7% of respondents are categorized as a particular group of CFL incorrectly for all datasets, and the highest proportion is 23.03% in 2009. The geographical mapping figures reveal the wide variation of the inconsistent CFL across different states over the 4 years. Notably, the results show that the highest proportion of population with incorrect CFL is 49.91% in South Carolina in 2009, and the lowest is 19.27% in Alaska in 2012. Furthermore, we investigate the impacts of CFL measured by the two methods on households’ stock market participation with Probit model. Comparing the marginal effects of the respondents’ CFL classified by the two methods, we discover that the respondents have a greater probability of holding stocks if they are categorized as the competence group by NA method in 2009. Additionally, if the respondents are categorized as the over-confidence group by NA method in 2012 and 2015, their probability of participating in stock market will be overestimated. Although the absolute difference of marginal effect of these CFL measured by the two methods is not great, it is important to attract enough attention to the methodology for measuring CFL.

The rest of this paper is organized as follows. Section 2 provides a detailed description of data source and study method. The results of empirical analysis and the discussions are presented in section 3, and section 4 provides the conclusions, limitations and future prospects of this study.

## Data and methodology

### Data source

This paper uses a representative survey data, i.e., NFCS 2009, 2012, 2015, and 2018.^4^ Data from multiple years contains more detailed information and insights on CFL and allows us to conduct comparative analysis. NFCS is funded by the U.S. Financial Industry Regulatory Authority (FINRA) Investor Education Foundation and conducted by Applied Research & Consulting. The survey has approximately 500 respondents per state, plus the District of Columbia, from June–October on each survey year. To provide additional utility for researchers working with the data, the NFCS 2015 included oversamples in four large states, for a total of 1,000 respondents each in California, Illinois, New York, and Texas. In contrast, NFCS 2018 included 1,250 respondents in Oregon and Washington. Findings from the survey are weighted to represent Census distributions, based on data from the American Community Survey. Therefore, to produce a reliable representation of the population as a whole, we use the state and nation weight to calculate the average of OFL and SFL at state and nation-level, respectively.

### The measurement of OFL

In all the four survey years, NFCS contains five basic questions to provide information about respondents’ OFL covering fundamental financial concepts encountered in daily life: compounding interest, inflation effect on the time value of money, the relationship between bond price and interest rate, interest payment difference on shorter and longer mortgages, and principle relating to diversification and risk. Although the five NFCS test items focus on basic financial knowledge in a simple question form, they have been found to be challenging for respondents and have been served as valuable indictors of OFL in many studies (e.g., Hastings et al., 2013; Lusardi and Mitchell, 2014; Allgood and Walstad, 2016).

Respondents can choose either they do not know the answer or refuse to answer, which can prevent respondents choosing at random. Specifically, the five financial questions in the OFL test include two options, ‘Do not know’ and ‘Prefer not to say.’ In line with previous research (Allgood and Walstad, 2013, 2016; Asaad, 2015; Porto and Xiao, 2016), a ‘1’ represents a correct response and a ‘0’ represents an incorrect response, a “Do not Know,” or a “Prefer not to say.” Table 1 summarizes the survey results of the five OFL questions in the U.S. for the year 2009, 2012, 2015, and 2018. The averages of each year demonstrate relatively low levels of OFL and imply that Americans have difficulty in applying financial decision-making skills to real-life situations. It is essential to mention that the OFL is decreasing over time. Therefore, although the five questions appear to be relatively simple, they are still challenging for many U.S. adults and can serve as reliable and valid indicators of OFL in the survey.

**Table 1:** OFL questions and the statistics of responses.

Questions	NFCS 2018	NFCS 2015	NFCS 2012	NFCS 2009
	Responses (Percentage)			
Interest: Suppose you had $100 in a savings account, and the interest rate was 2% per year. After 5 years, how much do you think you would have in the account if you left the money to grow?				
*More than $102*	72%	75%	75%	78%
Exactly $102	7%	8%	7%	6%
Less than $102	6%	5%	6%	5%
Do not know	13%	12%	11%	10%
Prefer not to say	1%	1%	1%	1%
Inflation: Imagine that the interest rate on your savings account was 1% per year and inflation was 2% per year. After 1 year, how much would you be able to buy with the money in this account?				
More than today	12%	10%	9%	7%
Exactly the same	10%	10%	9%	7%
*Less than today*	55%	59%	61%	65%
Do not know	21%	20%	20%	19%
Prefer not to say	1%	1%	1%	2%
Bond: If interest rates rise, what will typically happen to bond prices?				
They will rise	22%	19%	20%	18%
*They will fall*	26%	28%	28%	28%
They will stay the same	6%	5%	5%	5%
There is no relationship between bond prices and the interest rate	10%	9%	9%	10%
Do not know	36%	38%	37%	37%
Prefer not to say	1%	1%	1%	2%
Mortgage: A 15-year mortgage typically requires higher monthly payments than a 30-year mortgage, but the total interest paid over the life of the loan will be less.				
*TRUE*	73%	75%	75%	76%
FALSE	9%	8%	9%	9%
Do not know	17%	16%	15%	15%
Prefer not to say	1%	1%	1%	1%
Risk: Buying a single company’s stock usually provides a safer return than a stock mutual fund.				
TRUE	11%	10%	9%	6%
*FALSE*	43%	46%	48%	53%
Do not know	45%	44%	42%	40%
Prefer not to say	1%	1%	1%	1%
Objectively-measured FL mean at nation-level	2.7	2.83	2.88	2.99
Observations	27,091	27,564	25,509	28,146

Correct answers are italicized. The averages of OFL are calculated with the national weight.

### The measurement of SFL

The NFCS questionnaire also contains an alternative measure of overall FL to provide information about how respondents self-assess their own level of FL. In the survey, the self-assessment FL question is answered before the five test items of OFL to ensure that the self-rating is not affected by the objective answers.

Specifically, respondents in the NFCS survey are asked to assess their own level of financial knowledge on a 7-point Likert scale, whereby a ‘1’ reflects the lowest self-assessed level of financial knowledge and a ‘7’ reflects the highest level. Those choosing the answer ‘I do not know’ are excluded from our sample. This self-assessment question provides insights into how respondents perceive their own level of financial knowledge, and we label this as SFL. As shown in Table 2, a low percentage of respondents assess their FL level as below median, i.e., only 11, 9, 7, and 10% in 2009, 2012, 2015, and 2018, respectively. Whereas, a very high percentage of respondents assess their own FL level as above the median, i.e., 67, 73, 76, and 71% for 2009, 2012, 2015, and 2018, respectively. Most importantly, respondents, on average, rate their FL as 4.95, 5.15, and 5.24 in 2009, 2012, and 2015, respectively, which present an increasing trend of American’s financial confidence, but it is decreasing in 2018.

**Table 2:** SFL questions and the responses.

Questions	NFCS 2018	NFCS 2015	NFCS 2012	NFCS 2009
	Responses (Percentage)			
On a scale from 1 to 7, where 1 means very low and 7 means very high, how would you assess your overall financial knowledge?				
Low 1–3	10%	7%	9%	11%
Neutral 4	16%	14%	15%	18%
High 5–7	71%	76%	73%	67%
Do not know	2%	2%	2%	2%
Subjectively-perceived FL mean at nation-level	5.11	5.24	5.15	4.95
Observations	26,349	26,921	24,814	27,548

The averages of SFL are calculated with the national weight.

### The measurement of CFL

Similar to the previous studies, we divide respondents in each year into ‘OFL-high’ and ‘OFL-low’ groups using the individual OFL scores compared with OFL average scores (OFL-high: OFL > mean and OFL-low: OFL ≤ mean). The split of the sample in each year into “SFL-high” and “SFL-low” groups is based on the SFL scores and their averages in each year (SFL-high: SFL > mean and SFL-low: SFL ≤ mean). Like Porto and Xiao (2016), we categorize the sample into four CFL groups: OFL-high and SFL-high (defined as competence), OFL-low and SFL-low (defined as naivety), OFL-high and SFL-low (defined as under-confidence), and OFL-low and SFL-high (defined as over-confidence). In addition, we use the state subsample (SA method) and national sample (NA method) of each year to calculate the average scores of OFL and SFL,^5^ and then obtain two types of CFL for each respondent. The descriptive statistics of the two types of CFL are presented in the Section “The descriptive analysis of within-state average.”

## Results

### The descriptive analysis of within-state average

As shown in Table 3, the standard deviation of within-state average reveals a broader range of variation in OFL and in SFL across the 51 states. This finding means that there is a great difference of within-state average among states. Especially, the standard deviations of within-state OFL average for the four datasets are double those of SFL. Additionally, all the gaps between maximum and minimum of within-state OFL average for the four datasets excess 0.5. Given that the total OFL score is 5, above 10% difference of maximum and minimum and big deviation of within-state OFL average indicate that the residents’ OFL among states is more unbalanced than SFL.

**Table 3:** The statistics of within-state average.

	NFCS 2018		NFCS 2015		NFCS 2012		NFCS 2009	
	OFL	SFL	OFL	SFL	OFL	SFL	OFL	SFL
Mean	2.7	5.11	2.83	5.24	2.88	5.15	2.99	4.95
Min	2.46	4.97	2.53	5.08	2.53	5	2.75	4.78
Max	3.05	5.29	3.35	5.42	3.23	5.27	3.3	5.11
Std. Dev.	0.15	0.07	0.19	0.07	0.15	0.08	0.12	0.08

### The comparative analysis of the four CFL groups measured with the two methods

Based on SA method and NA method, we can obtain two types of CFL for each respondent. Table 4 summarizes the descriptive statistics of the two types of CFL. Compared with SA method, the NA method underestimates the over-confidence and naivety group while overestimates the under-confidence and competence group in most of the survey years. Specifically, the absolute gap of a certain CFL group measured with the two methods ranging from 1 to 5% includes all the four groups of 2015 dataset, competence, under-confidence and over-confidence group of 2018 dataset, competence and over-confidence group of 2012 dataset, and over-confidence group of 2009 dataset. In particular, the absolute gap of naivety and under-confidence group of 2012 dataset excesses 5% and naivety and competence group of 2009 dataset excesses 10%. However, only two groups, including naivety group of 2018 dataset and under-confidence group of 2009 dataset have the absolute gap below 1%. Considering the sample size over 20,000 for every year, even 1% absolute gap implies that there is a significant difference between the two types of CFL.

**Table 4:** Proportions of the four CFL groups measured with the two methods.

	NFCS 2018		NFCS 2015		NFCS 2012		NFCS 2009	
	NA method	SA method	NA method	SA method	NA method	SA method	NA method	SA method
Naivety	26.83%	26.74%	22.74%	26.73%	23.08%	28.67%	12.19%	22.21%
Competence	28.36%	29.70%	31.04%	28.61%	31.07%	27.95%	53.16%	38.29%
Under-confidence	31.85%	29.20%	33.37%	29.39%	35.18%	29.59%	17.23%	17.79%
Over-confidence	12.96%	14.36%	12.85%	15.28%	10.67%	13.79%	17.43%	21.71%
Observations	26,349		26,921		24,814		27,548	

### The distribution characteristics of state and population in the light of the two averages

Table 5 shows the number of states with a state FL average above or below the national average. As we know, if the within-state average of OFL (SAOFL) or SFL (SASFL) is higher than the national average of OFL (NAOFL) or SFL (NASFL), the relative level of OFL or SFL of respondents in a state will be underestimated by NA method and vice versa. The results in Table 4 show that the number of states with SAOFL above NAOFL is greater than the number of states with SAOFL below NAOFL. Such unbalance implies that the relative level of respondents’ OFL in most states could be overestimated using NA method. Comparatively, the relative level of respondents’ SFL is underestimated in most states.

**Table 5:** State distribution above the state and national average FL scores.

	NFCS 2018		NFCS 2015		NFCS 2012		NFCS 2009	
	OFL	SFL	OFL	SFL	OFL	SFL	OFL	SFL
The number of states with within-state average above national average	27	28	32	22	30	23	33	21
The number of states with within-state average below national average	24	23	19	29	21	28	18	30
Observations	51		51		51		51	

In addition, the higher proportion of the population above NAOFL than those above SAOFL in each year implies that if we use NAOFL as the comparative benchmark, the relative OFL level of respondents is overestimated, just as shown in Table 6. As for the relative SFL level, it is overestimated in 2009 but underestimated in 2018. According to these findings, we can infer that the competent population in NA method will be more than the same population in SA method, and the under-confident population in NA method will be less than the same population in SA method in 2009. But the under-confident population in NA method will be more than the same population in SA method in 2018. Similarly, we can get the conclusion that the population with over-confidence and under-confidence in NA method will be less than the same population in SA method. In contrast, the population with under-confidence and competency in NA method will be more in 2012 and 2015. These inferences are consistent with the results shown in Table 4.

**Table 6:** Population distribution above the state or national average.

	NFCS 2018		NFCS 2015		NFCS 2012		NFCS 2009	
	OFL	SFL	OFL	SFL	OFL	SFL	OFL	SFL
The proportion of the population above national average	60.21%	41.31%	64.41%	43.89%	66.25%	41.74%	70.38%	70.59%
The proportion of the population above within-state average	58.90%	44.06%	58.00%	43.89%	57.54%	41.74%	56.08%	60.00%
Observations	26,349		26,921		24,814		27,548	

### An explanation for the source of the difference between the two types of CFL

**Figure 1:**
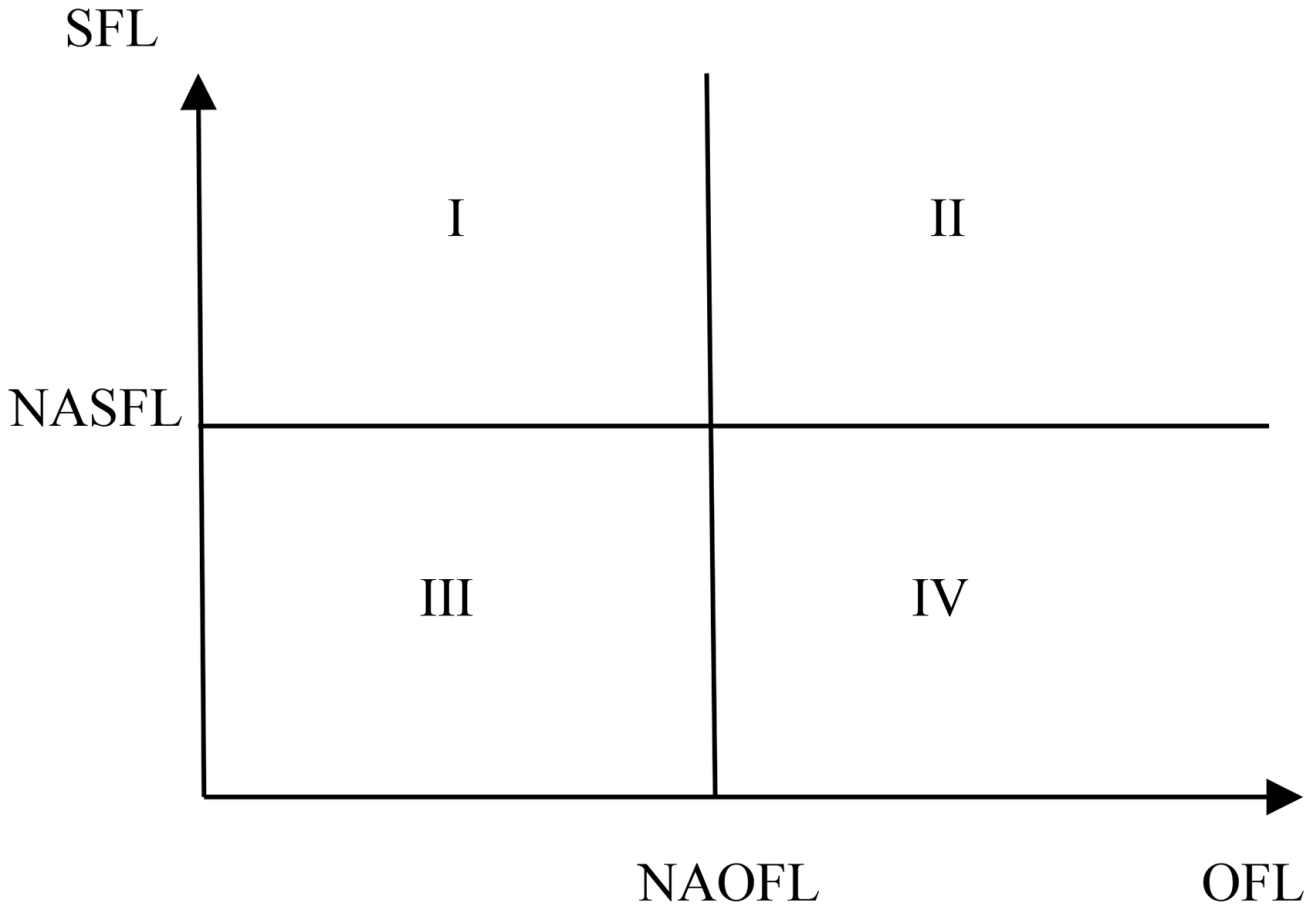
The four cases of states.

Using the NASFL and NAOFL as cutoff points, Figure 1 is constructed with four regions where each region represents a different case of states. In the first case, the state with lower SAOFL and higher SASFL will be located in region I, and the over-confidence group will be overestimated and the other groups will be underestimated by NA method. In the second case, the state with higher SAOFL and SASFL will be located in region II, and the competent group will be overestimated and the other groups will be underestimated by NA method. In the third case, the state with lower SAOFL and SASFL will be located in region III, and the naivety group will be overestimated and the other groups will be underestimated by NA method. In the fourth case, the state with higher SAOFL and lower SASFL will be located in region IV, and the under-confidence group will be overestimated and the other groups will be underestimated by NA method.

### Who will be categorized as the miscategorized group of CFL measured with NA method?

We argue that the CFL measured with SA method is more reasonable than NA method, and then create a binary variable, *Y*, to represent whether the respondents in the sample are incorrectly categorized. *Y* equals 1 if the CFL of a respondent calculated by the two methods are different, and equals to 0 otherwise. *Y* = 1 represents that the respondent is categorized in a wrong group of CFL by NA method. Table 7 reports the characteristics of variable *Y*, which shows that there are 10.70% of respondents categorized as a particular group of incorrect CFL in all the survey years. In particular, the largest proportion (23.03%) incorrectly categorized occurs in 2009, and the lowest proportion (4.06%) occurs in 2018.

**Table 7:** The characteristics of variable *Y.*

Characteristics	All datasets	NSCF 2018	NSCF 2015	NSCF 2012	NSCF 2009
Mean	0.11 (0.31)	0.04 (0.20)	0.06 (0.25)	0.09 (0.28)	0.23 (0.42)
The total number of respondents with *Y* = 1	11,303	1,070	1,726	2,162	6,345
The proportion of respondents with *Y* = 1	10.70%	4.06%	6.41%	8.71%	23.03%
Observations	1,05,632	26,349	26,921	24,814	27,548

The standard deviation is given in parenthesis.

**Figure 2:**
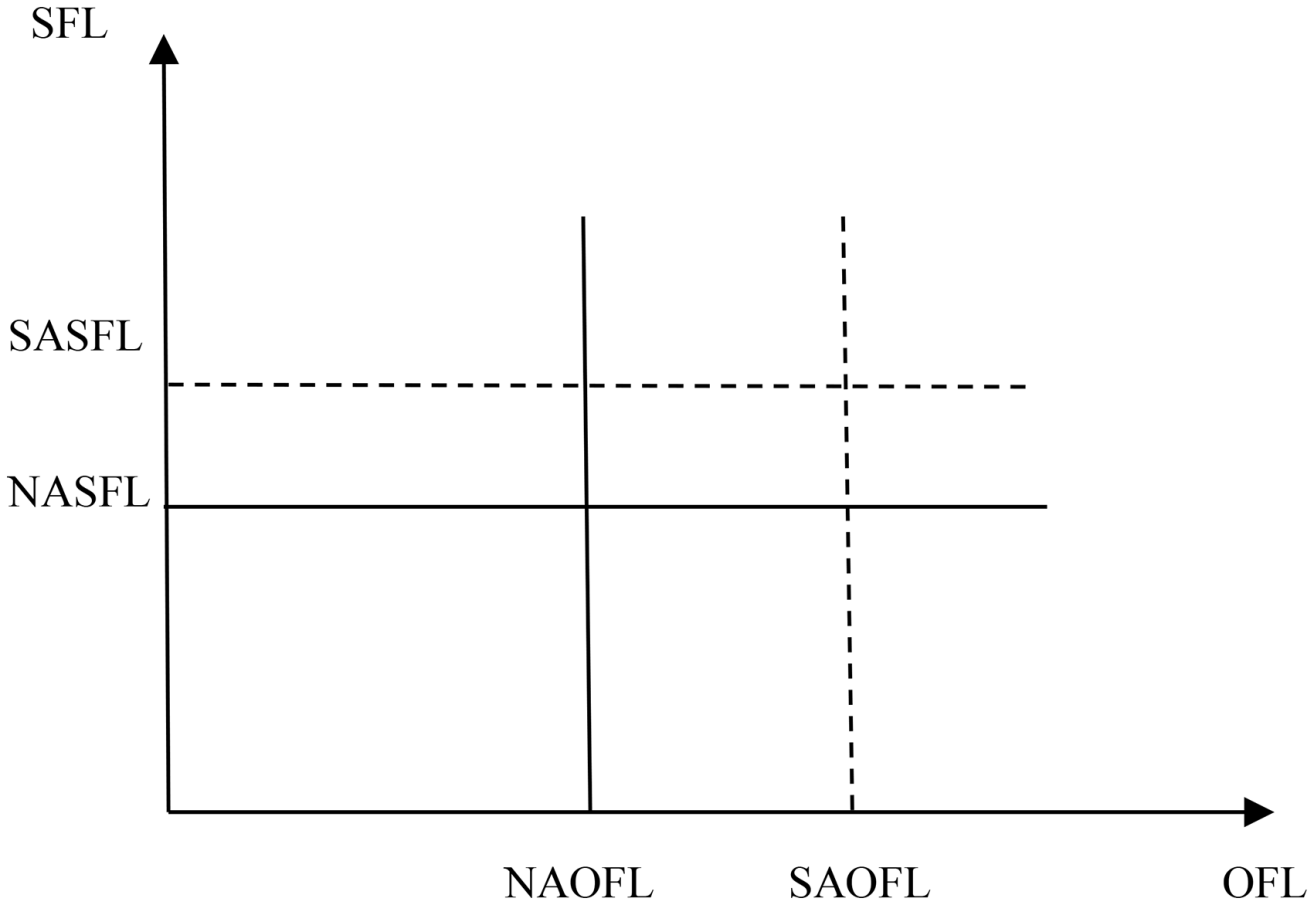
The comparison of state and national averages.

Taking the region II in Figure 1 for example, we further discuss who will be categorized as a wrong group of CFL by NA method. As shown in Figure 2, if the CFL of a respondent locates between the solid line and the dotted line, he/she will be miscategorized. That is to say, the OFL and SFL scores of those who are categorized as a wrong group of CFL by NA method should meet anyone of the following two conditions.min (*SAOFL*, *NAOFL*) < OFL score < max (*SAOFL*, *NAOFL*)min (*SASFL*, *NASFL*) < SFL score < max (*SASFL*, *NASFL*)

### Which states have respondents with *Y* = 1?

**Figure 3:**
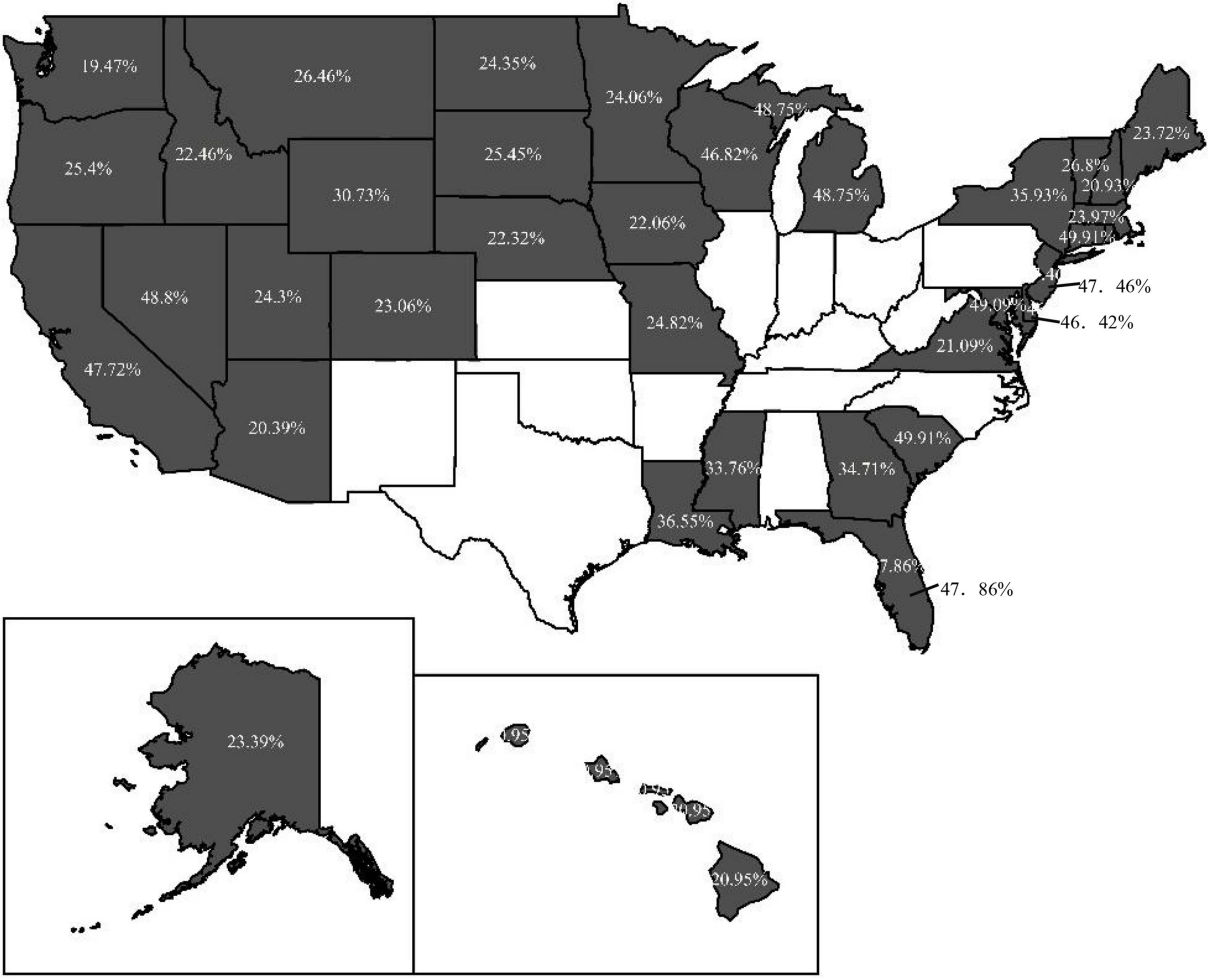
The distribution of the proportion of respondents with *Y* = 1 in 2009.

**Figure 4:**
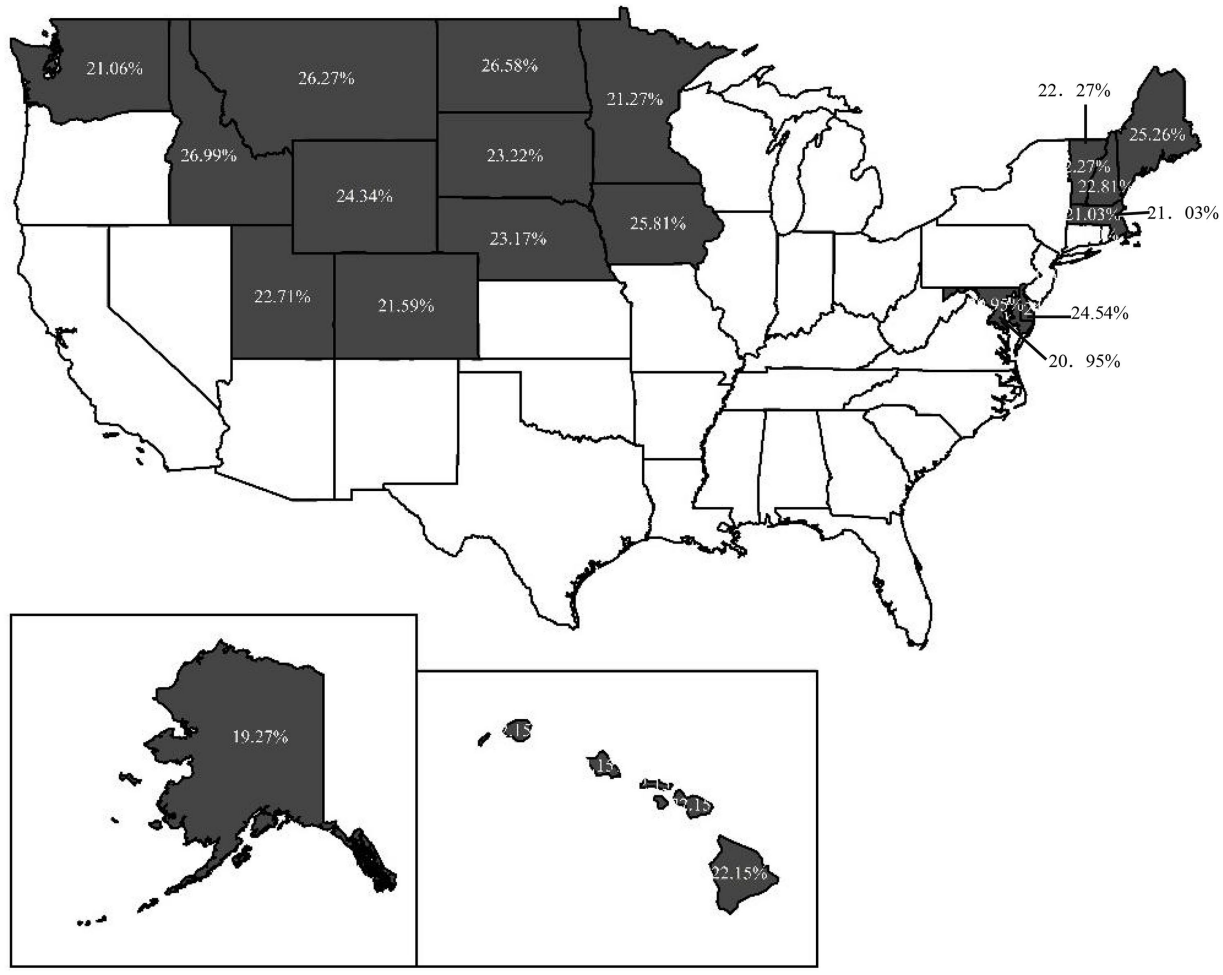
The distribution of the proportion of respondents with *Y* = 1 in 2012.

**Figure 5:**
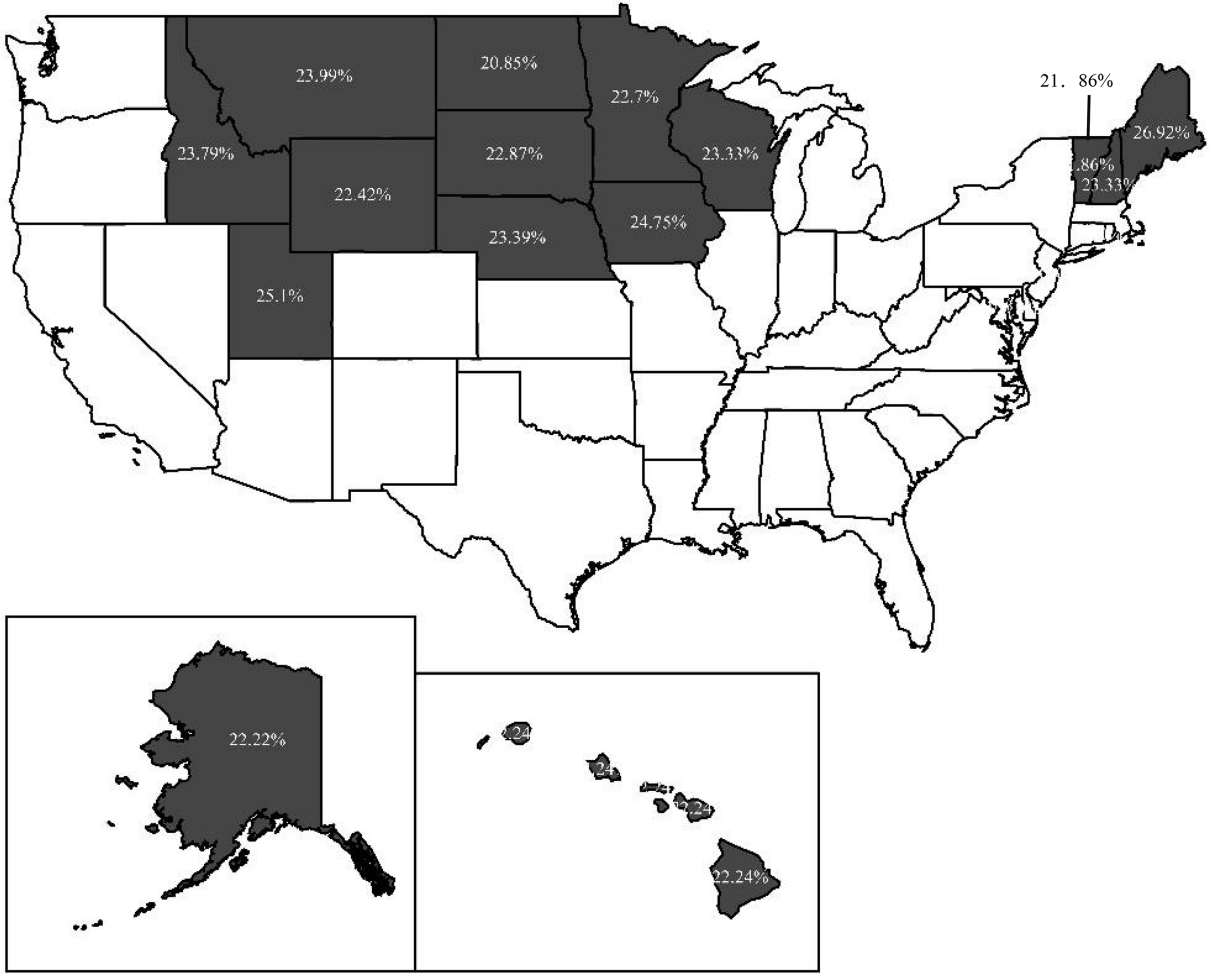
The distribution of the proportion of respondents with *Y* = 1 in 2015.

**Figure 6:**
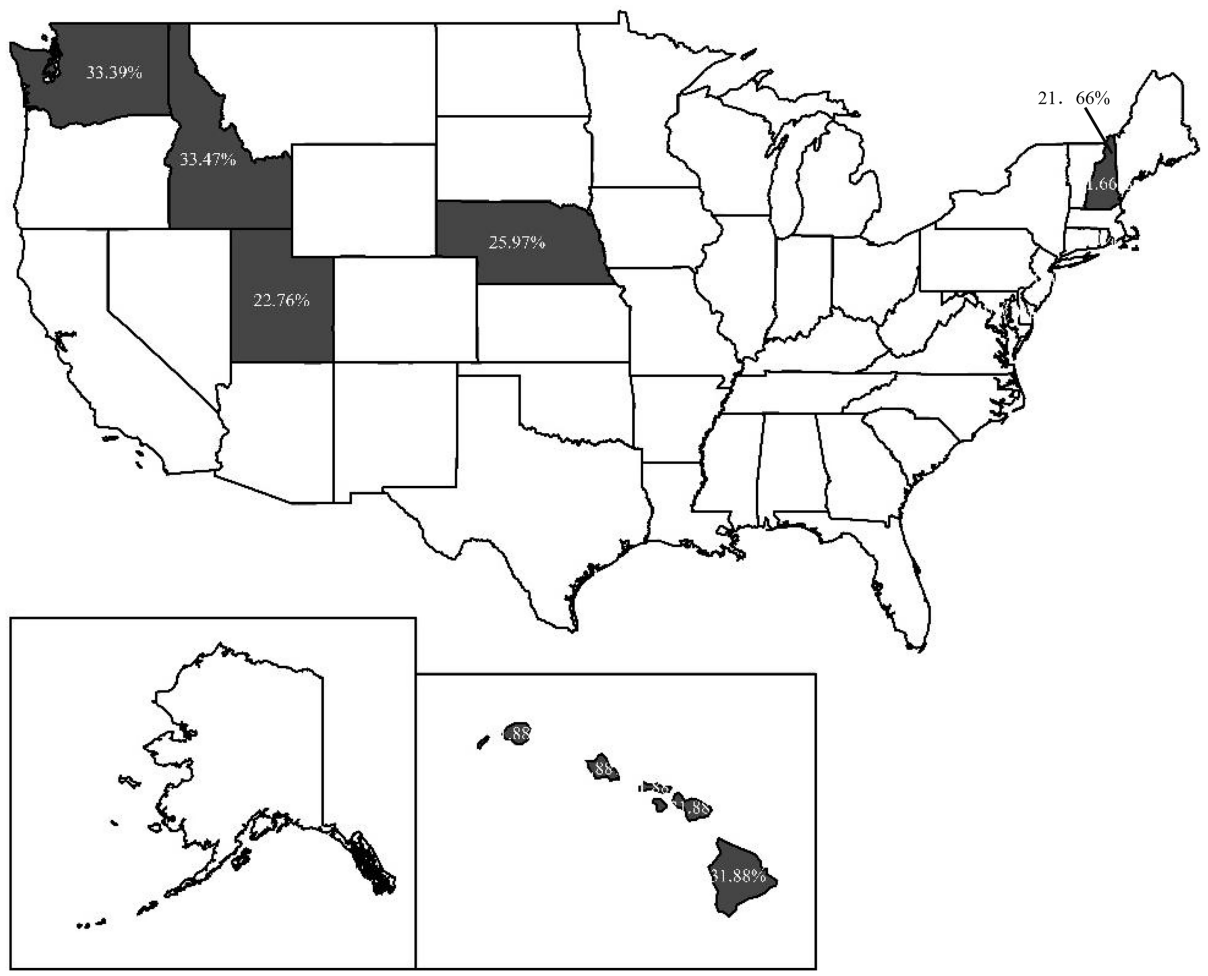
The distribution of the proportion of respondents with *Y* = 1 in 2018.

We use ArcGIS software to illustrate the distribution of the proportion of respondents with *Y* = 1 in states for the 4 years. Figures 3–6 indicate that 36, 19, 15, and 6 states have respondents with *Y* = 1, while the other states do not have any respondent miscategorized for 2009, 2012, 2015, and 2018, respectively. From 2009 to 2018, the lowest proportion of population miscategorized is 19.27% in Alaska in 2012, whereas the highest proportion is 49.91% in South Carolina in 2009. This finding means that once there are respondents miscategorized in a state, the proportion of population miscategorized is substantial. Therefore, if we use the national average as the comparative benchmark to measure CFL, the results will mislead our judge on the CFL of respondents in many states.

### The effects of the two types of CFL on financial behavior: A comparative analysis

We further provide a comparative analysis of the difference between the effects of the two types of CFL on financial behavior. Stock market participation is one of the essential financial behaviors of households,^6^ which contributes to family wealth accumulation. Therefore, we specify a Probit regression model and use it to estimate the different effects of the two types of CFL on stock market participation while controlling for the demographic characteristics of respondents, just as the study of Allgood and Walstad (2016). The Probit model is nonlinear regressions where coefficients are fitted with the maximum likelihood to the following function:


$$P\left(\mathrm{Stock market participation}=1\right)=\phi \left(\beta^{\prime }x\right)$$

Where *Φ* is the standard normal distribution, x is a vector of explanatory variables, and *β* is coefficient vector to be estimated. The datasets of 4 years provide weights to match Census distributions for age by gender, ethnicity, education, and Census division, and then we use the survey commands available in Stata to compute clustered robust standard error. The definitions of variables other than CFL are described in Table 8.

**Table 8:** The definition of demographic variables.

**Variables**	**Definition**
Stock market participation	Not including retirement accounts, households have any investment in stocks, bonds, mutual funds, or other securities.
Male	Male respondent.
Age	Respondent’s age in years.
Education	Education by groups:**<Highschool** = did not complete high school;** =Highschool** = high school graduate - regular high school diploma and high school graduate - GED or alternative credential; **Somecollege** = some college, **College** = college graduate and associate’s degree and bachelor’s degree; **Postgraduate** = post graduate degree.
White	White or Caucasian.
Marital status	**Married** = married; **Single** = single; **Divorced/sep** = divorced or separated; **Widow** = widow or widower.
Income	Respondent’s income.
Employment	**Selfemployed** = self-employed; **Full-time** = work full-time for an employer; **Part-time** = work part-time for an employer; **Homemaker** = homemaker; **Student** = full-time student; **Disabled** = permanently sick, disabled, or unable to work; **Unemployed** = unemployed or temporarily laid off; **Retired** = retired.
Children	The number of children who are financial dependents.
Living arrangements	**LiveAlone** = only adult in household; **LivePartner** = live with my spouse/partner/significant other; **LiveParents** = live in my parents’ home; **LiveOther** = live with other family, friends, or roommates.

Similar to Allgood and Walstad (2016), the six categorical variables for age (18–24, 25–34, 35–44, 45–54, 55–64, and 65+) are transformed into a continuous variable by setting the age at the mid-point of each range and at 70 for those respondents indicating their age was over 65. Income was represented by six categorical variables (<$15 K, $15-25 K, $25-35 K, $35-50 K, $50-75 K, $75-100 K, $100-150 K, and > $150 K) and it was transformed using the similar procedures for age variables. For lowest (<$15 K) or highest (>$150 K) income categories, income was set at the respective highest or lowest amounts. For other income categories, income was set to the mid-point of the range. The others are (0, 1) dummy variables.

For simplicity, Table 9 only reports the marginal effects of the two types of CFL on stock market participation and their differences. The results in Table 9 indicate that the combination of OFL and SFL significantly affect households’ probability of participating in the stock market for the four survey years and for the two methods. This finding is consistent with those of Allgood and Walstad (2016), who only use NFCS 2009 dataset and NA method to analyze the effects of perceived and actual financial literacy on several financial behaviors, including stock market participation behavior. However, we discover that the significant differences of the marginal effects of the CFL measured with the two methods on stock market participation occur in 2009, 2012 and 2015. Specifically, the competent respondents have a greater probability of holding stocks if we use NA method to measure the competence group in 2009. Similarly, if we use NA method to measure the over-confidence group in 2012 and 2015, its effect on stock market participation will be overestimated.

**Table 9:** The marginal effects of the two types of CFL on stock market participation and their differences.

CFL	NFCS 2018			NFCS 2015			NFCS 2012			NFCS 2009		
	NA method	SA method	Difference	NA method	SA method	Difference	NA method	SA method	Difference	NA	SA	Difference
										method	method	
Competence	0.200^***^	0.202^***^	1.02	0.182^***^	0.178^***^	1.59	0.165^***^	0.165^***^	0	0.157^***^	0.137^***^	*4.34* ^**^
	−23.38	−23.7	−0.313	−21.71	−22.06	−0.208	−19.03	−20.06	−0.955	−15.6	−17.25	*−0.037*
Under-confidence	0.077^***^	0.078^***^	0.26	0.080^***^	0.073^***^	2.5	0.061^***^	0.060^***^	0.04	0.043^***^	0.051^***^	0.48
	−9.28	−9.26	−0.611	−9.63	−9.09	−0.114	−7.13	−7.38	−0.84	−3.75	−5.73	−0.488
Over-confidence	0.158^***^	0.157^***^	0.03	0.164^***^	0.150^***^	*11.94* ^***^	0.127^***^	0.120^***^	*2.74* ^*^	0.095^***^	0.084^***^	1.03
	−16.29	−16.46	−0.855	−16.88	−16.58	*−0.001*	−11.92	−12.35	*−0.098*	−8.55	−9.71	−0.31
Observations	23,505			24,425			22,346			25,232		

The naivety is treated as the base group. The regressions are estimated with the weighted Probit method in Stata. Marginal effects of Probit regressions with an absolute value of robust *z*-statistic are in parenthesis. The last column of each survey year reports the Chi-squared statistics of a Wald test for the difference of marginal effects, and *p* values are in parenthesis. *, **, *** indicate significance at the 10, 5, and 1% level, respectively. The statistically significant differences are italicized.

## Conclusion

This paper provides a new method to measure the CFL and proves its reasonability through the comparative analysis with the NA method. We find the significant difference between the results of CFL of the two methods, and explain the cause of the difference. Our findings imply that if we use NA method to measure the CFL, a particular group of respondents will be miscategorized. Especially, the difference of the effects between the two types of CFL on stock market participation behavior indicates that if we use the imprecise measure of CFL to study the relationship between CFL and financial behaviors, the conclusions could mislead the practitioners and policymakers. Using the national average as a benchmark to determine all the respondents’ relative FL levels for different states is not meaningful, and will lose the practical appeal to tackle the regional inequalities of FL among the households. Therefore, we suggest that the within-state average of OFL and SFL, not the national average, should be taken as the comparative benchmark for the measure of CFL.

This study unveils that the comparative benchmark related to geographical factor really plays an important role in the measure of CFL. However, we only explore the different impacts of CFL measured with the two methods on stock market participation behavior. The other finical behaviors, such as retirement planning and investment decision, are also significantly affected by individuals’ CFL (e.g., Liao et al., 2022; Yeh and Ling, 2022; Seraj et al., 2022a,b). Therefore, it could be intuitive to check whether the CFL measured by the two methods can produce different marginal effects for other specific financial behaviors in a particular survey data. Additionally, some emerging research fields other than financial behaviors, such as sustainable performance of enterprise affected by the FL of entrepreneur (Alshebami and Murad, 2022; Seraj et al., 2022a, b), should further considered the impact of the CFL measured with our improved method.

## Data availability statement

Publicly available datasets were analyzed in this study. This data can be found at: https://finrafoundation.org/knowledge-we-gain-share/nfcs/data-and-downloads.

## Author contributions

FL: acquization of original data, software, formal analysis, methodology, and manuscript revision. HL: funding acquisition, reference management, and manuscript revision critically. WL and QY: conceptualization, writing – review and editing, methodology, and manuscript revision. FL and HL: data calculation and writing. All authors contributed to the article and approved the submitted version.

## Funding

The authors would like to acknowledge funding from the National Social Science Fund of China (Grant No. 21XJY018), the Social Science Fund of Xinjiang (Grant No. 20BJY04), the Major Counselor and Investigation Project of Xinjiang University of Finance and Economics (Grant No. 2022XZZ001), and the Social Science Fund of Jiangsu University of Technology (Grant No. KYY20512).

## Conflict of interest

The authors declare that the research was conducted in the absence of any commercial or financial relationships that could be construed as a potential conflict of interest.

## Publisher’s note

All claims expressed in this article are solely those of the authors and do not necessarily represent those of their affiliated organizations, or those of the publisher, the editors and the reviewers. Any product that may be evaluated in this article, or claim that may be made by its manufacturer, is not guaranteed or endorsed by the publisher.

## Footnotes

^1^The OFL score is derived from the number of correct answers to multiple-choice or true/false survey answered by respondents, which consist of objective questions. The SFL is a self-rating that reflects how confident respondents are about their own financial knowledge. Some Survey data, such as National Financial Capability Study (NFCS) of U.S., provide us with both an OFL and a SFL score.

^2^In section "The measurement of OFL," we describe the calculation method of OFL in detail.

^3^The measure method of CFL taking the within-state average as comparative benchmark is referred to as SA method, while the measure method of CFL taking the national average as comparative benchmark is referred to as NA method in this paper.

^4^These data can be freely downloaded on https://finrafoundation.org/knowledge-we-gain-share/nfcs/data-and-downloads.

^5^The average of OFL and SFL at state level are calculated with state weight, while the average of OFL and SFL at nation level are calculated with nation weight.

^6^Many studies have focused on the stock market participation behavior of household, such as van Rooij et al. (2011), von Gaudecher (2015) and Yeh and Ling (2022).
